# Customizably designed multibodies neutralize SARS-CoV-2 in a variant-insensitive manner

**DOI:** 10.3389/fimmu.2023.1226880

**Published:** 2023-08-10

**Authors:** Cecilia Abreu, Claudia Ortega, Natalia Olivero-Deibe, Federico Carrión, Aracelly Gaete-Argel, Fernando Valiente-Echeverría, Ricardo Soto-Rifo, Rafaela Milan Bonotto, Alessandro Marcello, Sergio Pantano

**Affiliations:** ^1^ Institut Pasteur de Montevideo, Montevideo, Uruguay; ^2^ Laboratory of Molecular and Cellular Virology, Virology Program, Institute of Biomedical Sciences, Faculty of Medicine, Universidad de Chile, Santiago, Chile; ^3^ Millennium Institute on Immunology and Immunotherapy, Santiago, Chile; ^4^ Laboratory of Molecular Virology, The International Centre for Genetic Engineering and Biotechnology (ICGEB), Trieste, Italy

**Keywords:** entry inhibitor, avidity, multi-target, protein design, Omicron, coronavirus, antibody, neutralization

## Abstract

The COVID-19 pandemic evolves constantly, requiring adaptable solutions to combat emerging SARS-CoV-2 variants. To address this, we created a pentameric scaffold based on a mammalian protein, which can be customized with up to 10 protein binding modules. This molecular scaffold spans roughly 20 nm and can simultaneously neutralize SARS-CoV-2 Spike proteins from one or multiple viral particles. Using only two different modules targeting the Spike’s RBD domain, this construct outcompetes human antibodies from vaccinated individuals’ serum and blocks *in vitro* cell attachment and pseudotyped virus entry. Additionally, the multibodies inhibit viral replication at low picomolar concentrations, regardless of the variant. This customizable multibody can be easily produced in procaryote systems, providing a new avenue for therapeutic development and detection devices, and contributing to preparedness against rapidly evolving pathogens.

## 1 Introduction

The COVID-19 pandemic continues to cause widespread morbidity and mortality worldwide (https://covid19.who.int). This scenario partly occurs because the protection provided by different immunization schemes against newer variants depends on previous infections, vaccination, and hybrid immunity (1, 2). This situation, added to the selective pressure exerted on the SARS-CoV-2 virus during the pandemic, has led to the sporadic appearance of variants that confer different degrees of resistance (3, 4).

Significant therapeutic efforts have been directed against the Spike (S) glycoprotein, which is exposed on the virion and infected cells surface (5–7), and uses the human angiotensin-converting enzyme 2 (hACE2) as its main cellular receptor (8). Upon binding to hACE2 via its receptor binding domain (RBD), S undergoes a cascade of protein cleavages and conformational changes leading to the fusion of the viral and cellular membranes (9).

Given the critical relevance of the S-ACE2 specific recognition for SARS-CoV-2 entry into the host cell, several inhibitors for this protein-protein interaction (hereafter called binders) have been developed as candidates for therapeutic intervention or as the base of detection devices (10–12). However, the continuous emergence of SARS-CoV-2 variants of concern (VOCs), characterized by mutations located in crucial positions of S, almost invariably reduced the neutralizing capacity of natural antibodies (3), nanobodies (13), and designed proteins (14–16). Therefore, there is a continuous demand for developing adaptable molecular scaffolds capable of efficiently blocking the entry of SARS-CoV-2 variants. From a more general perspective, preparedness to respond swiftly to new pathogens is paramount to countermeasure future outbreaks that compromise global health and economy.

The design of molecular architectures comprising multiple binders is a promising strategy to increase effective affinity (i.e., avidity) as multiple epitope targeting may help overcome the effect of viral escape mutants (17–21). However, the S protein’s topology poses severe challenges to designing effective multimeric scaffolds. Multiple copies are sparsely distributed on the virion’s surface, each featuring three RBDs that can alternate between differently exposed conformations with distances among main RBD epitopes varying from nearly one to ten nanometers (22).

Therefore, we undertook a computational chemistry approach to design a multimeric protein scaffold amenable to be decorated with arbitrary RBD binders with high potential for detection and therapeutical applications. Hence, we pursued a molecular design strategy to fulfill the following requirements: i) it can cope with the S conformational flexibility; ii) it maximizes the multiplicity of RBD binders with a molecular weight between 70 kDa and 150 kDa; iii) it can be easily produced; iv) it minimizes possible side effects as cytotoxicity or immunogenicity; v) it is thermostable; vi) it must be easily customizable, i.e., it should be possible to interchange the RBD binder modules without compromising any of the previous characteristics.

## 2 Materials and methods

### 2.1 Protein expression

Genes encoding the designed protein sequences were codon optimized for *E. coli* expression and synthesized by GenScript. Sequences were inserted into pT7-GFP (XL-1, binder A, and B) or pT7-SUMO (XL-2, binder A and binder C) vectors (23). Binder A and B are followed by GFP fusion protein and 8x-Histidine tag (His-tag), and XL-1 is followed by 8xHis-tag. A 6xHis-tag, SUMO fusion protein and a TEV cleavage sequence precedes XL2 and binder C. Plasmids were then transformed into chemically competent *E. coli* BL21 (XL-1, binder A and B) and SHuffle T7 (binder A and C) cells (New England Biolabs).

Protein expression was performed using the 2YT media supplemented with 100 μg/ml ampicillin. After the cell culture reached an optical density of 0.8-1, protein expression was induced with 0.1 or 0.5 mM IPTG for 20 hours at 20°C. The cells were harvested by centrifugation at 4,000 *g* for 20 min., and then resuspended in buffer A (300 mM NaCl, 50 mM Tris-HCl, pH 7.5) supplemented with 50 µg/mL lysozyme (Sigma) and EDTA-free protease inhibitors cocktail (Roche). The cell suspension was subjected to sonication, and the debris was removed by centrifugation at 18,000 *g* for 40 minutes at 4°C. The clarified extract was applied to a 1 ml HisTrap column (Cytiva), and upon washing with Buffer A with 20 mM imidazole, the His-tagged protein was eluted with Buffer A containing 500 mM imidazole. SUMO-HisTag fusion was cleaved from XL2 and binder C protein by adding TEV-HisTag protease at a 1:40 molar ratio. The sample was dialyzed against buffer A for 12h at 4°C and then applied to 1 ml HisTrap column (Cytiva) to remove the SUMO-HisTag, TEV-HisTag, and uncleaved protein. The proteins were further polished by a Superdex G-200 size exclusion chromatography column (10/300, GE Healthcare) run in buffer A. All protein samples were characterized on a 10% SDS-PAGE, obtaining a purity higher than 95%. Protein concentrations were determined by absorbance at 280 nm using predicted extinction coefficients.

The Recombinant Protein facility at Institut Pasteur de Montevideo produced all RBD variants. Briefly, proteins with an Avitag-HisTag fusion were expressed in Expi293F cells and purified using HisTrap columns. When needed, the proteins were biotinylated *in vitro*.

### 2.2 Cells and viruses

Huh7 cells expressing hACE2 (Huh7-hACE2) (24) were obtained from the American Type Culture Collection (ATCC). HEK-293T cell line was obtained from HIV Reagent Program (NIH), and HEK-293T cells expressing hACE2 (HEK-293T-hACE2) were obtained from BEI Resources, NIAID, NIH (NR-52511) (surface binding inhibition) or previously generated by us (25). All cell lines were cultured in Dulbecco’s modified Eagle’s medium (DMEM, ThermoFisher, Paisley, UK) supplemented with 10% fetal bovine serum (FBS, ThermoFisher, Paisley, UK). Working strains of SARS-CoV-2 ICGEB-FVG_5 (26) (ancestral strain with D614G mutation) and the Omicron variant SARS-CoV-2 BA.1_4 isolated in Trieste, Italy, were routinely propagated and titrated as described elsewhere (24).

### 2.3 ELISA

96-well plates (Medisorp, Thermo Fisher Scientific) were coated with 0.2 µg/well of the RBD variants in PBS buffer overnight at 4°C and incubated for one hour at 37°C with 200 μl of blocking solution (PBS-1% bovine serum albumin (BSA)). Subsequently, the binding of antibodies present in serum from Pfizer twice-vaccinated individuals (final dilution 1:500, BEI Resources, NIAID, NIH: Pooled Human Serum Sample, Pfizer Vaccine, NRH-17727) was evaluated in the absence or presence of different concentrations of XL-1 and XL-2 molecules (from 1.6 nM to 136 nM). For this purpose, 100 μl of the serum/molecules in blocking solution were added and incubated for 1 hour at 37°C. The plates were washed thoroughly with PBS-0.1% Tween20. For detecting the IgG antibodies that remained bound to the RBD, a rabbit anti-human IgG peroxidase conjugate (P021402-5, Dako) was applied, followed by an incubation step with the peroxidase substrate 3,3’,5,5´-tetramethylbenzidine (TMB) (Promega) for 15 minutes. The reaction was stopped by adding 11% H_2_SO_4_. The absorbance was then determined at 450 nm.

### 2.4 Cell-surface binding inhibition assay

We preincubated 36 nM of RBD alone (positive control) or with different amounts of the molecules (from 1 to 1088 nM) for one hour at 37°C in a 96 v-bottom well plate in 50 µl of binding buffer (PBS,1% BSA, 2 mM EDTA). Then, 50 µl of binding buffer containing 1x10^5^ HEK-293T-hACE2 cells were added and incubated for one hour on ice. Three washes were performed centrifuging at 400 g for three minutes and resuspending in 150 µl of binding buffer. Finally, the supernatant was removed, and 30 μl of a 1:400 dilution of Strep-Tactin-Dye649 (IBA Lifesciences GmbH) in binding buffer were plated and incubated 30 min. at room temperature. After six washes, as previously described, cells were acquired on an Accuri C6 (BD Bioscience) cytometer, and the results were analyzed using the FlowJo™ v10 Software (BD Life Sciences). The mean fluorescence intensity (MFI) of duplicate positive controls and negative controls (cells without RBD) were averaged and considered as 100% of surface binding and background, respectively. Thus, the percentage of surface binding inhibition of each molecule dilution was calculated as the complement of the division between the corresponding MFI and the positive control MFI after subtracting the background. All conditions were run in duplicate, and the standard deviation was calculated. The results were corroborated in two or three independent experiments. This method was adapted in-house from (27).

### 2.5 Production of an HIV-1-based SARS-CoV-2-Spike pseudotyped virus

Pseudotyped viruses carrying SARS-CoV-2 Spike Wuhan (D614G), Delta, or Omicron variants were produced as described in (25). Briefly, HEK-293T cells were co-transfected with the HIV-1 proviral vector pNL4.3-ΔEnv-Luciferase and the corresponding pCDNA-SARS-CoV-2 Spike coding vectors at a 3:2 ratio using PEI.

Spike codifying vectors were purchased from GenScript and designed to lack the last 19 amino acids of the C-terminal end (SΔ19), to avoid retention at the endoplasmic reticulum. Spike sequences contained the following mutations over the Wuhan reference strain: B lineage (D614G), Delta lineage B.1.617.2, and Omicron lineage BA.1.

At 48 hours post-transfection, pseudotypes were recovered from the supernatant, cleared by centrifugation at 850 g for 5 minutes at room temperature, diluted in 50% fetal bovine serum (Sigma-Aldrich), aliquoted and stored at -80°C until use. Pseudoviruses were quantified by HIV-1 Gag p24 Quantikine ELISA Kit (R&D Systems) following the manufacturer’s instructions.

### 2.6 Pseudotyped virus neutralization assay

Different amounts of the molecules (from 100 nM to 0.003 nM) were 2-fold serially diluted in supplemented DMEM. Fifty μL of each dilution were incubated with 3 ng of HIV-1-based SARS-CoV-2 variants in triplicate for 1 hour at 37°C, generating a final dilution from 50 nM to 0.0015 nM. Then, 1x10^4^ HEK-ACE2 cells were added to each well. HEK-ACE2 cells incubated in the absence of pseudotyped viruses were used as a negative control, whereas HEK-ACE2 cells incubated with pseudotypes viruses in the absence of molecules were used as positive controls. Cells were lysed 48 hours later, and firefly luciferase activity was measured using the Luciferase Assay Reagent (Promega) in a Glomax 96 Microplate luminometer (Promega). Relative luminescence units (RLUs) of positive controls were averaged and considered as 0% neutralization, while RLUs measured in negative controls were considered as 100% neutralization. Thus, the percentage of neutralization of each molecule dilution was calculated as the complement of the division between the corresponding RLUs and the RLUs obtained at the positive controls after subtracting the background. This calculation was done independently for each technical replica and for each spike variant. The results were corroborated in independent experiments.

### 2.7 Neutralization assay

The proteins XL-1 and XL-2 were prepared in serial dilutions at a range concentration of 25 – 0.006 nM. SARS-CoV-2 Wuhan (D614G) and Omicron were prepared at a multiplicity of infection 0.1 and pre-incubated with the proteins in their respective dilutions for 1 hour at 37°C. 96-well plates containing monolayers of Huh7-hACE2 were washed with PBS and incubated with 180 μl of cells’ medium containing virus treated at the various concentrations. The drug 17Y was included as positive control at 20 μM (28). Plates were incubated for 48 hours at 37°C and fixed with 4% PFA (Paraformaldehyde, Sigma). Then, cells were treated with the recombinant anti-Spike monoclonal antibody (CR3022) (29) or with a nucleocapsid commercial monoclonal antibody (Cat. No. 40588-T62, Sino Biological Inc, Beijing, China) and appropriate fluorescent secondary antibodies. Images were acquired using Operetta (PerkinElmer, Waltham, ZDA), and the total number of cells and the number of infected cells were analyzed using the Columbus Image Data Storage and Analysis System (PerkinElmer). The results were corroborated in two independent experiments.

### 2.8 Half-maximal inhibitory or effective concentration (IC50 or EC50)

IC50 and EC50, defined as the concentration of the molecule yielding a 50% diminution of mean fluorescence, luciferase activity, or infected cells compared to the negative and positive controls, were calculated by modeling a four-parameter non-linear regression with variable slope using GraphPad Prism v9.1.2 (La Jolla, California, USA).

### 2.9 Modeling

AlphaFold2 models were generated using the Google Colab web server (30). The sequence alignment shown in **Supplementary Material** was performed with ESPript 3.0 (31).

## 3 Results

### 3.1 Molecular design

After an exhaustive literature search, we selected the C-terminal segment of the mouse Cartilage Matrix Protein (CMP) (32). Although we cannot rule out immunogenicity in humans, the murine version was preferred as it would facilitate preclinical tests in mice. Nevertheless, immunogenicity studies go beyond the scope of this study.

Molecular modeling suggested that removing the steric hindrance created by the N-terminal region would favor the formation of tetramers and pentamers (**Figure S1A**). Therefore, the CMP’s C-terminal segment provided a promising oligomerization scaffold to be decorated with suitable RBD binders. To rule out possible steric hindrances among RBD binders, we fused the flexible and protease resistance linkers reported by Kim et al. (33) at the N- and C-termini of the oligomerization scaffold. Combining this molecular architecture with nanobody-sized binder modules results in proteins of nearly 30 kDa, which facilitates its production in procaryote systems.

**Figure 1:**
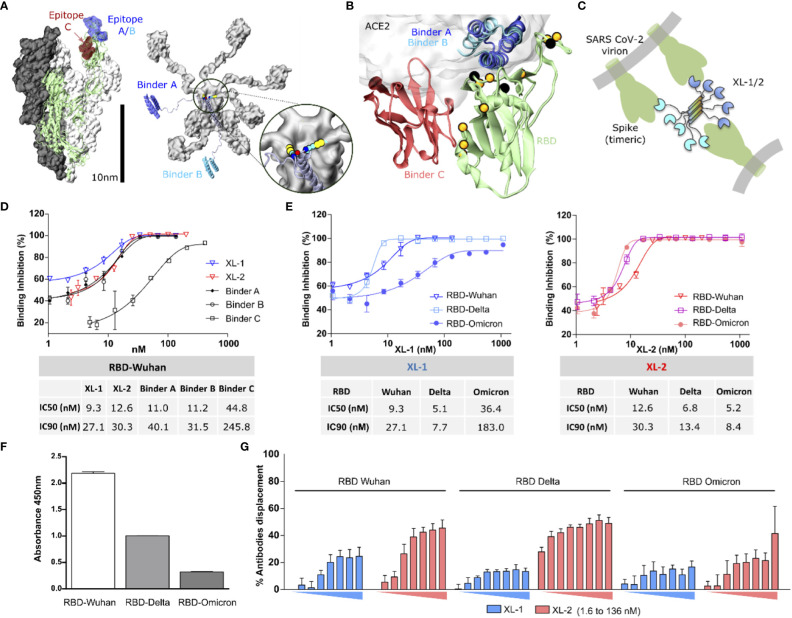
Molecular representation of the construct and RBD binding capacity. **(A)** Left: Molecular representation of the soluble region of the Spike protein as determined in the PDB structure 7JZL. One of the monomers is shown in cartoon representation (green), with the epitopes on the RBD highlighted in blue (epitopes A/B) and red (epitope C). Right: Molecular model of a Xiang-Liu 1 molecule featuring binders A and B at the extremities (blue and light blue, respectively). Both figures are drawn at the same scale to emphasize the possibility of Xiang-Liu binding more than one Spike trimer. The inset shows the cysteine residues forming the disulfide bridges at the extremity of the five-helix bundle. **(B)** Cartoon representation of an RBD molecule (green) with binders A (blue), B (light blue), and C (red). The semitransparent surface indicates the position of hACE2. Black spheres indicate the position of mutations reported for variants before Omicron, while those in orange correspond to Omicron. **(C)** Schematic representation of a Xiang-Liu construct illustrating possible interaction modes with different trimeric spikes on the same virion or neighboring viral particles. **(D)** Dose-response cell surface binding inhibition curves of Wuhan RBD in the presence of isolated binders and Xiang-Liu molecules. Calculated IC50 and IC90 are shown in the table below. **(E)** Dose-response cell surface binding inhibition curves in the presence of different RBDs from Wuhan, Delta, and Omicron. Analogous curves for isolated binders are shown in **Figure S3**. Calculated IC50 and IC90 are shown in the table below. **(F)** RBD variant recognition by antibodies from SARS-CoV-2 post-immunization human serum. Immunoassay immobilizes different RBDs VOCs and revealing the binding antibodies with HRP-conjugated anti-human IgG. **(G)** Percentage of antibodies displacement upon increasing concentrations of Xiang-Liu molecules (from 1.6 nM to 136 nM) calculated from ELISA experiments immobilizing different RBDs VOCs and revealing the binding antibodies from human post-immunization SARS-CoV-2 serum with HRP-conjugated anti-human IgG. We show the average value among two replicates and their standard deviation in all cases.

Moreover, the proteins spontaneously pentamerize into “multibodies”, linked by disulfide bridges (inset in **Figure 1A**). To the best of our knowledge, this is the maximum multiplicity reported for a mammalian scaffold of these dimensions with a molecular weight of nearly 150 kDa. Hence, it maximizes avidity, fulfilling all the criteria mentioned above. **Figure 1A** illustrates the gross structural determinants of this construct, which size is comparable to that of the S protein. We named this scaffold Xiang-Liu, after the voracious, multiheaded Chinese mythology snake.

### 3.2 Multibodies inhibit RBD-ACE2 binding on the cell surface

To test the versatility of Xiang-Liu, we selected three thermostable RBD binders reported in the literature to target two of the most immunogenic regions of the RBD (34). Binders A and B correspond to *in-silico* designed miniproteins with 3D structures deposited under the codes 7JZM and 7JZU in the Protein Data Bank (PDB), respectively (**Figure S1B**) (22). Both binders bind to the same epitope on the RBD (**Figure 1B**). Binder C corresponds to the camelid nanobody whose 3D coordinates are reported as chain B in the PDB structure 7OLZ (**Figure S1B**) (19). Unlike the previous binder modules, binder C recognizes specifically a spatially separated region on the RBD surface (**Figure 1B**). These three binders displayed low nM to pM affinity against the Wuhan variant. We designed two versions named Xiang-Liu 1 and 2 (hereafter referred to as XL-1 and XL-2). XL-1 features the binders A and B at the N- and C-termini of the multimerization domain, while in XL-2, we replace binder B with binder C (**Figures 1B**, **S1C**). Because of their global dimensions and the flexible linkers used to connect the binders to the pentamerization scaffold, the Xiang-Liu constructs may bind simultaneously to one or more RBDs within a trimeric S protein, to RBDs belonging to different spikes on the surface of a single virion or even bridge the surfaces of neighboring virions (**Figure 1C**).

According to our modeling hypotheses, both constructs form a pentameric array stabilized by disulfide bridges (**Figure S2**). Therefore, we sought to compare the performance of the isolated binders and the Xiang-Liu constructs to interfere with the ACE2-RBD interactions in different assays.

To provide a proof-of-concept of the effectiveness of our scaffold to overcome the effects of spontaneously arising mutations, we first tested its neutralizing capacity against the SARS-CoV-2 ancestral variant, for which the binder modules were originally designed. Moreover, we also tested it against Delta and Omicron variants, which achieved worldwide distribution and contained point mutations in the RBD protein-protein binding interface.

Cell surface binding inhibition assays showed that the three isolated binder modules and the XL constructs could inhibit the binding of soluble (monomeric) RBD to hACE2 exposed on the cell surface. Isolated binders A and B, and both Xiang-Liu constructs showed inhibiting concentrations at low nM concentrations, while binder C inhibits the binding only in the mid nM range (**Figure 1D**). Regardless, molarity calculation suggests that complete binding inhibition is achieved at roughly stoichiometric concentrations of soluble RBD and any of the binder modules in the solution. Therefore, IC90 values could better gauge our constructs’ actual inhibitory capacity.

Testing XL-1 and XL-2 resulted in a limited gain in cell surface binding inhibition capacity against the Wuhan RBD, in particular, when compared against the isolated binders A and B (**Figure 1D**). This result is not unexpected since RBDs are present as isolated protein modules in this *in-vitro* assay (unlike in the S trimer). On the other hand, the low nanomolar affinities of binders result in a purely diffusional bottleneck for the interaction.

However, the cell surface binding inhibition capacity of the monoparatopic XL-1 decreased against newer variants (**Figure 1E**). Indeed, much higher concentrations of XL-1 were insufficient to achieve full displacement of Omicron’s RBD. In contrast, the biparatopic XL-2 showed minimal sensibility to the different variants, showing an IC90 of around 10 nM for all cases tested (**Figure 1E**). This result contrasts with the behavior of the isolated modules that showed a markedly reduced capacity to displace the affinity against the Omicron’s RBD from the ACE2 protein present in the cell membrane (**Figure S3**).

### 3.3 Displacement of human antibodies from vaccinated individuals

Because of the high affinity observed *in vitro*, we speculated that the Xiang-Liu constructs should be able to outcompete natural antibodies. Thus, we asserted the capacity of our multibody to displace human antibodies elicited upon vaccination. ELISA plates were coated with RBD modules corresponding to the different variants and incubated with pooled human serum from twice-inoculated individuals (BEI Resources, see Methods). In agreement with previous reports (35), we observed a progressive decrease in the recognition by serum antibodies against newer variants (**Figure 1F**). Subsequently, we added XL-1 and XL-2 and measured the antibodies displaced in each case. Coincubation with XL-2 results in over two-fold higher displacement than XL-1 (**Figure 1G**), as could be expected because of the biparatopic nature of this construct.

We could displace nearly 50% of the antibodies bound to the RBDs at the maximal concentrations used. Hence, it seems reasonable to speculate that we displaced all the antibodies bound to the two targeted epitopes. If so, nearly 50% of the elicited antibodies recognize peripheral regions of the RBD outside the epitopes targeted by our binders. Therefore, multibodies decorated with epitope-specific binders might constitute a promising strategy to characterize structural footprints of the immune response.

### 3.4 Multibodies inhibit pseudovirus entry in a variant-insensitive manner

**Figure 2:**
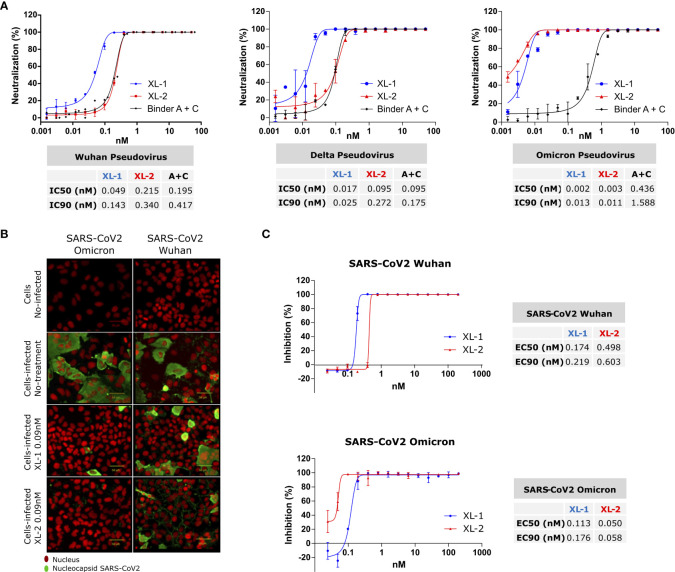
SARS-CoV-2 Neutralization and Inhibition. **(A)** Neutralization assay on pseudotyped SARS-CoV-2 particle variants adding XL-1 and XL-2 multibodies. Additionally, equivalent concentrations of binders A and C were added to estimate the avidity gain provided by the pentameric constructs. Calculated IC50 and IC90 are shown in the tables below. **(B, C)** Infection inhibition of SARS-CoV-2 infective viruses with XL-1 and XL-2. **(B)** Representative image of infected Huh7-hACE2 cell, the nucleus in red and SARS-CoV2 nucleocapsid in green. **(C)** Virus infection inhibition with different concentrations of XL-1 and XL-2 molecules.

Despite the convenience of the *in-vitro* assay to test different variants and conditions, fully exploiting the avidity of Xiang-Liu multibodies requires the presence of an intact, trimeric S protein. To address this issue, we generated pseudotyped viruses carrying either the SARS-CoV-2 S protein from Wuhan, Delta, or Omicron variants, as previously reported by us (3). Assays using the pseudotyped virus carrying the Wuhan S revealed a higher neutralization by XL-1 (IC90 = 0.143nM) compared to XL-2 (IC90 = 0.34nM, **Figure 2A**). The lower affinity of binder C in XL-2 can readily explain this. Indeed, repeating the experiment with an equimolar mixture of the isolated binding modules A and C results in a similar neutralization capacity than XL-2 (**Figure 2A**). Thus, the binding affinity of Binder A for the RBDs of the ancestral and Delta variants dominates the specific recognition.

Considering the marked decrease in the inhibitory capacity of the isolated binders against the Omicron’s RBD (**Figure S3**), a reduction in the neutralizing capacity against Omicron pseudoviruses would be expected for all the constructs. Strikingly, the neutralization assay performed on that variant showed a better performance of XL-2 and an increase in the neutralization capacity of both constructs (XL-1_IC90 = _0.013nM, XL-2_IC90 = _0.011nM).

Therefore, the avidity conferred to Xiang-Liu by the decameric array of binders overcomes the loss in the affinity of the individual binders against the Omicron variant in the trimeric context of the full-length S protein.

### 3.5 Inhibition of viral infection

Finally, we measured the neutralization capacity using a High-Content Assay protocol recently described (24).

**Figure 2B** shows that pre-incubation of XL-1 and XL-2 with SARS-CoV-2 viral particles prevents the virus from binding to the receptor and entering the target cell. Moreover, a virucidal drug used as positive control produced similar results on the viral (**Figure S4**) (28).

The dose-dependent inhibition curves of XL-1 and XL-2 with intact viruses follow the same trend observed with pseudotyped viruses (**Figures 2A, C**). In particular, XL-2 showed a higher sensitivity against Omicron with an EC_90_ of 0.058 nM, compared with the EC_90_ of 0.176 nM observed for XL-1. Therefore, the data presented here provide compelling evidence about the capacity of our heterotypic multibodies to cope with viral variability, keeping or improving the neutralizing ability in a wide variety of physicochemical contexts.

## 4 Discussion and conclusions

During the pandemic, the scientific community has devoted significant effort to developing new antiviral strategies based on innovative molecular designs. Noteworthy, therapeutical efforts directed against the protease or polymerase seem to be almost insensitive to viral variability (36). Notwithstanding, the spontaneous appearance of escape variants continues to challenge the effectiveness of molecular approaches blocking Spike’s RBD.

Different “mosaic” based protein designs have been reported, including tandem repeats, dimerization/multimerization domains, decorated vesicles or nanoparticles (37). Some multimeric o tandem architectures achieve avidities similar to that avidity obtained from the biparatopic architecture of the Xiang-Liu construct (38, 39). However, we are not aware of another customizable mammalian protein scaffold able to present ten binding modules spanning nearly 20 nm in space and with a molecular weight compatible with renal clearance.

Higher avidity can be achieved by decorating vesicles or nanoparticles, but assembling such structures may be expensive or intricate (37). As our construct can be expressed in procaryote systems and pentamerizes spontaneously, we hope it will provide a convenient and cost-effective platform for further research.

Beyond its inhibitory power, the extreme avidity of Xiang-Liu could also prove highly valuable to define antibody footprints on specific epitopes using simple setups as the competition ELISAs shown in **Figure 1G**.

Clearly, a limitation of this study is the relatively small number of variants tested. However, the large number of mutations introduced by Omicron in relation to the original and Delta variant underlines the capacity of Xiang-Liu to cope with the viral variability.

A possible point of concern regards the ability to decorate the pentameric scaffold with arbitrary binding modules. Nevertheless, binders B, and C in XL-1 and XL-2 display a significantly different folding (**Figure S1B**). This suggests that the Xiang-Liu scaffold can be decorated with a large variety of possible binders. While this remains to be tested for each particular case, structural considerations suggest that binder modules in the nano body-size range should not present steric hindrances, keeping similar characteristics to those shown here.

The constructs reported here are biparatopic, i.e., decorated with two different binders at the N- and C- termini. Building multiparatopic pentamers, although possible, may face technical issues, as controlling the proportion of given constructs in the pentamers could be technically challenging.

In this study, we used the murine version of CMP, which will eventually facilitate preclinical studies in mice, although it could be potentially immunogenic in humans. We intended to provide a proof-of-principle of the neutralizing capacity of the Xiang-Liu scaffold against different SARS-CoV-2 variants. Therefore, immunogenicity studies go beyond the scope of this work.

Nevertheless, the CMP family is highly conserved among mammals (**Figure S4**). Hence, even if the present scaffold could potentially present immunogenicity problems, it seems safe to speculate that substituting the pentamerization motif with that of the human CMP would not alter the properties of the construct. Indeed, predictions performed with Alphafold2 multimer provided identical results to those shown in **Figure S1A**.

Besides its potential against SARS-CoV-2, the versatility of the Xiang-Liu multibody architecture may contribute to our preparedness against new pandemic events as it can be easily adapted to arbitrary targets from other pathogens. The simplicity of producing new high-affinity nanobodies against arising variants or even the cost-effective and reliable in-*silico* optimization of protein-protein interfaces (40), might allow for a swift response against emerging variants of concern.

## Data availability statement

The raw data supporting the conclusions of this article will be made available by the authors, without undue reservation.

## Author contributions

Experimental design, protein production, cell surface binding inhibition, ELISA assays, and drafting of the manuscript: CA. Setting up of the cell surface binding inhibition assay and production of RBD variants: CO and NO-D. Biochemical experiments: FC. Neutralization assays: AG-A, RS-R, and FV-E. Infectivity assays: RM and AM. Designed molecular scaffold, conceptualization, drafting of the manuscript, and acquiring the funding: SP. All authors reviewed and accepted the final version
