# Supplementary material for: Customizably designed multibodies neutralize SARS-CoV-2 in a variant-insensitive manner
Source: Front Immunol. 2023 Aug 10;14:1226880. doi: 10.3389/fimmu.2023.1226880 (PMC10447908; doi:10.3389/fimmu.2023.1226880)
Supplement: Supplementary file 1 [file Image_1.pdf]

Supplementary Material

Customizably designed multibodies neutralize SARS-CoV-2 in a variant-insensitive manner

Cecilia Abreu[1], Claudia Ortega[1], Natalia Olivero-Deibe[1], Federico Carrión[1], Aracelly Gaete-Argel[2,3], Fernando Valiente-Echeverría[2,3], Ricardo Soto-Rifo[2,3], Rafaela Milan Bonotto[4], Alessandro Marcello[4] and Sergio Pantano[1*]

[1]Institut Pasteur de Montevideo, Montevideo, Uruguay, [2]Laboratory of Molecular and Cellular Virology, Virology Program, Institute of Biomedical Sciences, Faculty of Medicine, Universidad de Chile, Santiago, Chile, [3]Millennium Institute on Immunology and Immunotherapy, Santiago, Chile, [4]Laboratory of Molecular Virology, International Centre for Genetic Engineering and Biotechnology (ICGEB), Trieste, Italy.

*Correspondence: Sergio Pantano, Email: spantano@pasteur.edu.uy

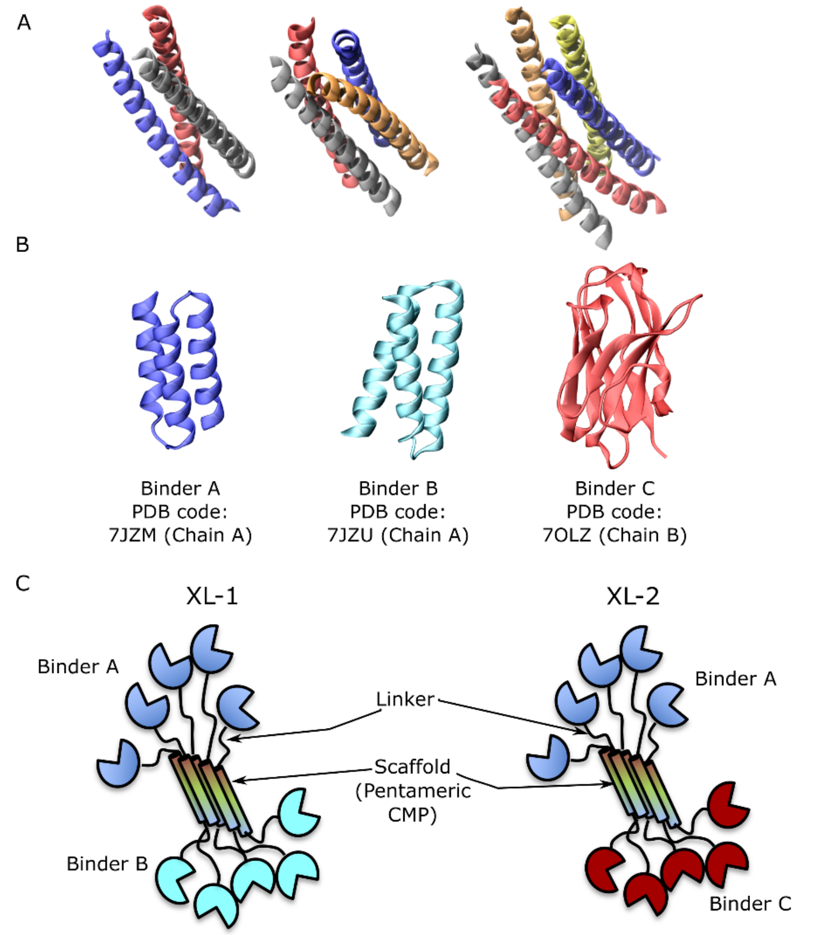

Supplementary Figure 1. A) To gain insights into the possible oligomerization states of the Cterminal domain of Cartilage Matrix Protein (CMP), we performed structural predictions based on artificial intelligence (Alphafold2) using the C-terminal helix of CMP. We obtained trimers, tetramers, and pentamers. Attempts to predict hexamers or higher oligomers result in combinations of trimers, tetramers, and pentamers. Free energy calculations performed using the Prodigy server (https://wenmr.science.uu.nl/prodigy/) suggest that tetramers and pentamers are more favorable than trimers by nearly 2 kcal/mol. **B)** Cartoon representation of the RBD binders used in the Xiang-Liu constructs and their respective naming and Protein Data Bank codes. **C)** Schematic representation of the pentamers XL-1 and XL-2.

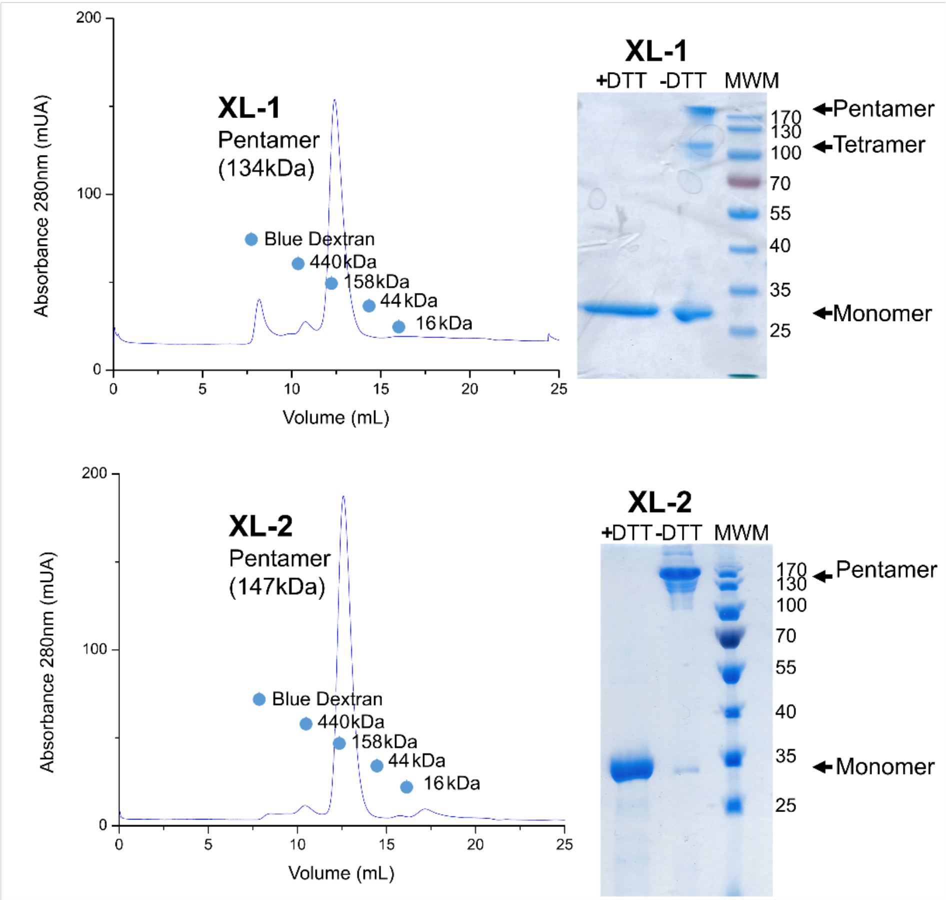

Supplementary Figure 2. Size exclusion chromatography of XL-1 and XL-2 in a Superdex 200 10/300 column. The column calibration data is shown in blue dots. The main peak was analyzed by SDS-PAGE 10% stained with Coomassie Blue. MWM: molecular weight marker, +DTT, and -DTT: samples under reducing and non-reducing conditions, respectively, indicating the relevance of the disulfide bridges for the presence of multimeric species.

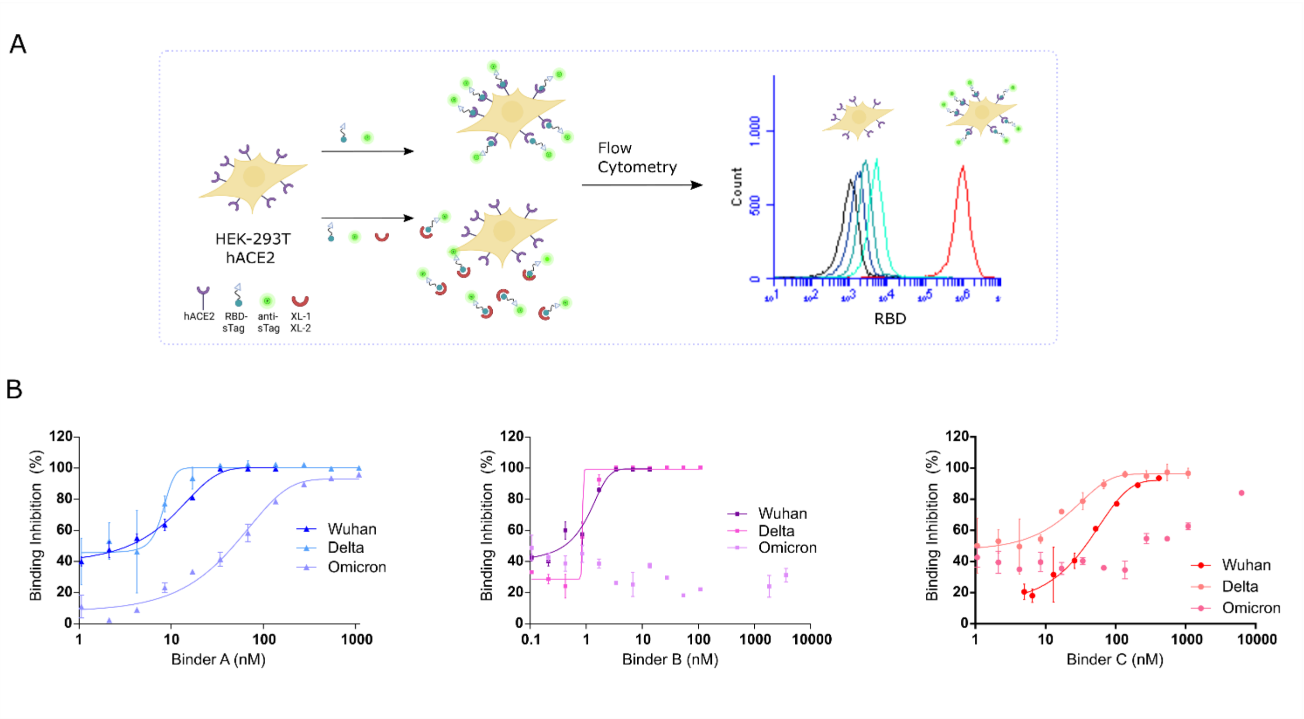

Supplementary Figure 3. **A)** Schematic representation of the cell surface binding inhibition method: binders are first incubated with tagged RBD. Subsequently, HEK-293 cells overexpressing hACE2 are added to the solution, and the displacement of RBD from hACE2 is measured by cell cytometry as a shift in the mean fluorescence intensity. **B)** Dose response cell surface binding inhibition curves for binders A, B, and C against different RBD variants.

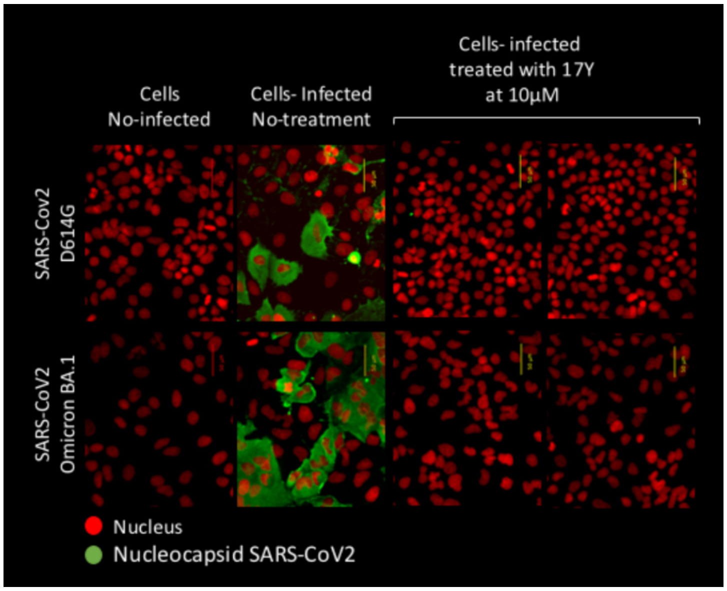

Supplementary Figure 4. Virucidal control experiment for the infection assay. Representative image of infection assay of Huh7-hACE2 cell, the nucleus in red and SARS-CoV2 nucleocapsid in green. The data reported in the two left columns is identical to that in Figure 2B. In the right panels, the virucidal compound 17Y, previously reported by us (28) is used as a positive control for the inhibition of infection.

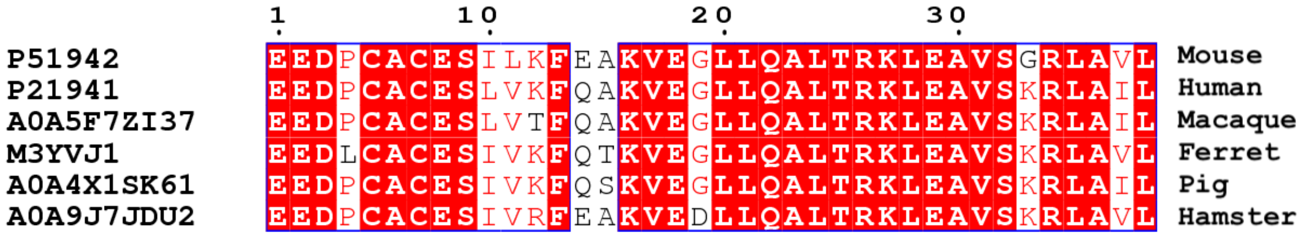

Supplementary Figure 5. Sequence alignment of the multimerization domain of Cartilage matrix proteins of selected mammals indicated on the right. The UniProt code of each sequence is reported on the left column.
